# Restrictive filling pattern of transmitral inflow in acute decompensated heart failure with preserved ejection fraction: insights from the KCHF registry

**DOI:** 10.1136/openhrt-2026-004093

**Published:** 2026-04-17

**Authors:** Yutaro Miyoshi, Takao Kato, Takeshi Morimoto, Neiko Ozasa, Hidenori Yaku, Yasutaka Inuzuka, Yodo Tamaki, Erika Yamamoto, Yusuke Yoshikawa, Takeshi Kitai, Moritake Iguchi, Kazuya Nagao, Yuichi Kawase, Takashi Morinaga, Yutaka Furukawa, Kenji Ando, Yukihito Sato, Koichiro Kuwahara, Koh Ono, Takeshi Kimura

**Affiliations:** 1 Kyoto University Graduate School of Medicine Department of Cardiovascular Medicine, Kyoto, Kyoto Prefecture, Japan; 2 Department of Data Science, Hyogo Medical University, Nishinomiya, Hyogo Prefecture, Japan; 3 Department of Heart Failure and Transplantation, National Cerebral and Cardiovascular Center, Suita, Osaka, Japan; 4 Cardiology, Shiga Medical Center for Adults, Moriyama, Japan; 5 Cardiology, Tenri Hospital, Tenri, Japan; 6 Department of Cardiology, National Hospital Organization Kyoto Medical Center, Kyoto, Japan; 7 Department of Cardiology, Osaka Red Cross Hospital, Osaka, Japan; 8 Department of Cardiology, Kurashiki Central Hospital, Kurashiki, Okayama Prefecture, Japan; 9 Department of Cardiology, Kokura Memorial Hospital, Kitakyushu, Fukuoka Prefecture, Japan; 10 Department of Cardiovascular Medicine, Kobe City Medical Center General Hospital, Kobe, Japan; 11 Department of Cardiology, Hyogo Prefectural Amagasaki General Medical Center, Amagasaki, Hyogo Prefecture, Japan; 12 Department of Cardiovascular Medicine, Shinshu Daigaku Daigakuin Igakukei Kenkyuka Igakubu, Matsumoto, Nagano, Japan; 13 Cardiology, Hirakata Kosai Hospital, Hirakata, Japan

**Keywords:** Heart Failure, Diastolic, HEART FAILURE, Echocardiography

## Abstract

**Background:**

The restrictive filling pattern of transmitral inflow has been shown to be associated with a poor prognosis in patients with heart failure (HF) with reduced ejection fraction or myocardial infarction. We aimed to investigate the significance of restrictive filling pattern in patients with HF with preserved ejection fraction (HFpEF).

**Methods:**

Among 4056 patients with acute decompensated HF in the Kyoto Congestive Heart Failure registry, we analysed 830 patients with HFpEF who had transmitral inflow data available in echocardiography. Patients whose early to late diastolic transmitral flow velocity (E/A ratio) ≥2 were classified as having a restrictive filling pattern of transmitral inflow. The main outcome measures were all-cause death and HF hospitalisation at 1 year.

**Results:**

Among 830 patients, a restrictive filling pattern was observed in 144 (17.3%) patients, who had higher prevalence of a history of atrial fibrillation and supranormal left ventricular ejection fraction (>65%), and higher brain natriuretic peptide level at discharge. The cumulative 1-year incidence of HF hospitalisation was significantly higher in patients with restrictive filling pattern than those without (31.0% vs 18.1%, p<0.001), while the cumulative 1-year incidence of all-cause death was not different between the two groups (14.2% vs 14.4%, p=0.93). After adjusting for confounding factors, the excess risk of restrictive filling pattern relative to non-restrictive filling pattern remained significant for HF hospitalisation (HR=1.58, 95% CI 1.10 to 2.27, p=0.01), but not for all-cause death (HR=0.92, 95% CI 0.60 to 1.42, p=0.70).

**Conclusions:**

A restrictive filling pattern of transmitral inflow was associated with an increased risk for HF hospitalisation, but not for all-cause death, in patients with acute decompensated HFpEF.

**WHAT IS ALREADY KNOWN ON THIS TOPIC**

- The restrictive filling pattern of transmitral inflow, E/A ratio ≥2, has been shown to reflect elevated left atrial pressure and be associated with a poor prognosis in patients with heart failure with reduced ejection fraction (HFrEF) or myocardial infarction, but there are no reports on the clinical prognosis of patients with restrictive filling pattern in heart failure with preserved ejection fraction (HFpEF).

**WHAT THIS STUDY ADDS**

- In patients with acute decompensated HFpEF, restrictive filling pattern of transmitral inflow in echocardiography was associated with an increased risk of heart failure hospitalisation.
- Patients with supranormal left ventricular ejection fraction (>65%) exhibited a higher frequency of restrictive filling pattern, suggesting that diastolic dysfunction may be a characteristic finding of heart failure with supranormal ejection fraction.

**HOW THIS STUDY MIGHT AFFECT RESEARCH, PRACTICE OR POLICY**

- Restrictive filling pattern is associated with clinical prognosis in patients with HFpEF, as well as in patients with HFrEF.
- Clinicians may consider paying attention to transmitral inflow while adhering to guideline-recommended algorithm for estimating diastolic function.

## Introduction

 Heart failure with preserved ejection fraction (HFpEF) is defined as HF with left ventricular ejection fraction (LVEF) ≥50%.^1 2^ HFpEF constitutes approximately half of all HF patients, and the proportion of HFpEF among all HF patients has been increasing in recent years.^3 4^ HFpEF is a heterogeneous phenotype that sometimes can be caused by specific cardiovascular diseases such as atrial fibrillation (AF) or coronary artery disease, or sometimes can be involved with non-cardiovascular diseases, such as obesity, skeletal muscle weakness, systemic inflammation or renal dysfunction.^5 6^

The essential elements of the diagnosis of HFpEF are clinical symptoms of HF and diastolic dysfunction.^7 8^ Diastolic dysfunction is strongly associated with elevated left ventricular (LV) filling pressure or left atrial pressure (LAP), and there have been many reports on the non-invasive estimation of diastolic dysfunction using transthoracic echocardiography (TTE). According to conventional guidelines, in cases of HF with reduced or mildly reduced ejection fraction (HFrEF or HFmrEF) or HFpEF with myocardial diseases, the transmitral inflow pattern has been well known to reflect LAP.^9^ On the other hand, in a patient with HFpEF, it has been reported that there was only weak correlation between transmitral inflow pattern and LV filling pressure.^10 11^

Recently, the new algorithm for estimating LV filling pressure in patients with normal LVEF by TTE has been proposed.^12 13^ When LAP is judged to be increased in the algorithm, early to late diastolic transmitral flow velocity, E/A ratio <2 and ≥2 are classified as grade 2 (mild to moderate increased LAP) and grade 3 (marked increased LAP) diastolic dysfunction, respectively. The restrictive filling pattern of transmitral inflow, E/A ratio ≥2, has been shown to be associated with a poor prognosis in patients with HFrEF or myocardial infarction,^14 15^ while there are no reports on the clinical prognosis of patients with restrictive filling pattern in HFpEF. Therefore, this study aimed to clarify the background and clinical prognosis of patients with restrictive filling pattern in HFpEF using the multicentre observational cohort study of Japanese patients with acute decompensated HF hospitalisation.

## Methods

### Study population

The Kyoto Congestive Heart Failure (KCHF) registry is a physician-initiated, prospective, observational, multicentre cohort study that enrolled the consecutive patients hospitalised for acute HF for the first time between 1 October 2014 and 31 March 2016, at 19 secondary and tertiary hospitals in Japan. The details of the KCHF registry have been described previously.^16 17^ Briefly, we consecutively enrolled the patients with acute decompensated HF defined by the modified Framingham criteria who were admitted to the participating centres and who received treatment for HF involving intravenous drugs within 24 hours after hospital presentation. Among 4056 patients enrolled in the KCHF registry, we excluded 271 patients who died during the index hospitalisation, 57 patients without follow-up data after discharge, 89 patients without baseline LVEF data, 1046 patients with AF or atrial flutter (AF or AFL) at TTE, and 569 patients without transmitral inflow data. In the remaining 2024 patients, we further excluded 1194 patients with HFrEF or HFmrEF. Finally, the current study population consisted of 830 patients with HFpEF and available transmitral inflow data, who were discharged alive.

### Definitions

All patients underwent two-dimensional and Doppler echocardiographic evaluation in each participating centre according to the latest guidelines at that time.^18^ The data from the earliest TTE after hospitalisation was used for analysis in this study. Transmitral inflow was evaluated using colour Doppler echocardiography in the apical four-chamber view and E/A ratio was calculated by peak E-wave velocity divided by peak A-wave velocity. According to the current guidelines for the evaluation of diastolic function by TTE, we classified patients whose E/A≥2 as restrictive filling pattern.^12^ LVEF was measured using the biplane modified Simpson’s method. We measured and assessed other parameters of TTE according to the guidelines,^18^ including LV end-diastolic diameter (LVEDD), LV end-systolic diameter (LVESD), left atrial diameter (LAD), tricuspid regurgitation peak gradient (TRPG), moderate or severe tricuspid regurgitation (TR), moderate or severe mitral regurgitation (MR), moderate or severe aortic stenosis (AS). The detailed definitions of other baseline patient characteristics are provided in online supplemental appendix 1.

### Outcomes

The main outcome measures in the current study were all-cause death and HF hospitalisation at 1 year. Other secondary outcome measures were cardiovascular death and non-cardiovascular death at 1 year. Death was regarded as cardiovascular in origin unless obvious non-cardiovascular causes could be identified. Cardiovascular death included HF-related death, sudden death, stroke-related death and death from other cardiovascular causes. Sudden death was defined as unexplained death in a previously stable patient. Stroke included either ischaemic or haemorrhagic stroke that required hospitalisation with symptoms lasting more than 24 hours. HF hospitalisation was defined as hospitalisation due to worsening of HF requiring intravenous drug therapy. The detailed definitions of other clinical outcome measures were described previously.^16^ All endpoint events were adjudicated by a clinical event committee.

### Statistical analysis

Categorical variables were presented as numbers and percentages. Continuous variables were presented as mean with SD or median with IQR according to their distributions. Categorical variables were compared using the χ^2^ test or Fisher’s exact test. Continuous variables were compared using the Student’s t-test or Wilcoxon rank-sum test based on their distributions. Continuous variables were dichotomised using clinically meaningful reference values or median values (table 1).

**Table 1:** Patient characteristics with and without restrictive filling pattern

HFpEF (N=830)	Restrictive filling pattern (N=144)	Non-restrictive filling pattern (N=686)	P value	N of patients analysed
Baseline characteristics				
Age, years	82 (73–87)	83 (75–88)	0.15	830
Age ≥80 years*	82 (56.9)	419 (61.1)	0.40	830
Women*	74 (51.4)	396 (57.7)	0.17	830
BMI, kg/m^2^	22.7 (20.6–25.1)	22.5 (20.1–25.4)	0.72	786
BMI (kg/m^2^) <22*	53 (38.4)	294 (45.4)	0.16	786
Obesity (BMI (kg/m^2^) ≥27.5)	14 (10.1)	97 (15.0)	0.18	786
Aetiology				
Coronary artery disease	23 (16.0)	104 (15.2)	0.80	830
Acute coronary syndrome*	5 (3.5)	45 (6.6)	0.18	830
Cardiomyopathy	8 (5.6)	30 (4.4)	0.51	830
Hypertensive heart disease	56 (38.9)	256 (37.3)	0.78	830
Valvular heart disease	35 (24.3)	173 (25.2)	0.92	830
Surgery during hospitalisation	3 (2.1)	12 (1.8)	0.73	830
Others	22 (15.3)	123 (17.9)	0.55	830
History				
Hypertension*	112 (77.8)	559 (81.5)	0.30	830
Diabetes*	50 (34.7)	240 (35.0)	1.0	830
Dyslipidaemia	63 (43.8)	268 (39.1)	0.30	830
Prior HF hospitalisation*	56 (38.9)	194 (28.3)	0.02	830
Prior PCI or CABG	29 (20.1)	144 (21.0)	0.91	830
Prior myocardial infarction*	20 (13.9)	89 (13.0)	0.79	830
Prior stroke*	22 (15.3)	116 (16.9)	0.71	830
Prior AF or AFL*	66 (45.8)	166 (24.2)	<0.001	830
Chronic lung disease*	19 (13.2)	106 (15.5)	0.61	830
Malignancy	11 (7.6)	114 (16.6)	0.005	830
Dementia	21 (14.6)	131 (19.1)	0.24	830
Chronic kidney disease	55 (38.2)	297 (43.3)	0.27	830
Daily life activities				
Current working	13 (9.0)	64 (9.3)	1.0	830
Not good adherence	17 (11.8)	105 (15.3)	0.30	830
Current smoker*	16 (11.2)	74 (11.0)	0.88	819
Ambulatory	115 (80.4)	512 (75.3)	0.23	823
Living alone*	28 (19.4)	149 (21.7)	0.58	830
Medications at admission				
Loop diuretics	65 (45.1)	293 (42.7)	0.64	830
MRA	24 (16.7)	80 (11.7)	0.13	830
ACEI/ARB	62 (43.1)	349 (50.9)	0.10	830
CCB	53 (36.8)	340 (49.6)	0.006	830
β-blockers	46 (31.9)	235 (34.3)	0.63	830
Aspirin	41 (28.5)	230 (33.5)	0.28	830
Anticoagulants	41 (28.5)	117 (17.1)	0.002	830
Warfarin	21 (14.6)	72 (10.5)	0.19	830
DOACs	20 (13.9)	45 (6.6)	0.006	830
NSAIDs	8 (5.6)	55 (8.0)	0.39	830
Clinical signs at presentation				
Systolic BP, mm Hg	153±37	159±38	0.08	829
Systolic BP<90 mm Hg*	2 (1.4)	15 (2.2)	0.75	829
Heart rate, /min	91±29	87±26	0.16	826
Heart rate <60 bpm*	16 (11.2)	85 (12.5)	0.78	826
NYHA III/IV	120 (84.5)	595 (86.9)	0.50	827
Sinus rhythm	85 (59.0)	526 (76.7)	<0.001	830
AF/AFL	46 (31.9)	105 (15.3)	<0.001	830
Laboratory tests at admission				
BNP, pg/mL	467 (256–858)	562 (311–1071)	0.18	707
Hb, g/L	116±23	108±22	<0.001	829
Anaemia*	95 (66.0)	530 (77.4)	0.006	829
Alb, g/dL	3.55±0.52	3.41±0.52	0.004	801
Alb <3.0 g/dL*	16 (11.4)	122 (18.5)	0.05	801
Na, mEq/L	139.5±4.0	139.4±4.2	0.71	829
Na <135 mEq/L*	13 (9.0)	83 (12.1)	0.32	829
K, mEq/L	4.1±0.6	4.2±0.7	0.07	829
K≥5 mEq/L	13 (9.0)	86 (12.6)	0.26	829
eGFR, mL/min/1.73 m^2^	45.0 (30.7–66.2)	42.3 (27.9–58.0)	0.16	829
eGFR <30 mL/min/1.73 m^2^*	34 (23.6)	203 (29.6)	0.16	829
Clinical signs at discharge				
Systolic BP, mm Hg	119±17	122±19	0.06	820
Systolic BP <114 mm Hg	65 (45.8)	244 (36.0)	0.04	820
Heart rate, /min	67.1±12.2	69.3±12.5	0.05	817
Heart rate <70/min	89 (63.1)	371 (54.9)	0.08	817
Sinus rhythm	100 (69.9)	543 (80.6)	0.007	817
AF/AFL	30 (21.0)	78 (11.6)	0.004	817
Residual PND	5 (3.6)	30 (4.5)	0.82	804
Residual orthopnoea	5 (3.6)	22 (3.3)	0.80	806
Residual DOE	38 (27.0)	170 (25.7)	0.75	802
Residual wheeze	7 (5.0)	27 (4.1)	0.64	802
Residual oedema	26 (18.3)	91 (13.7)	0.19	805
Residual jugular oedema	18 (12.7)	34 (5.2)	0.002	799
Loss of appetite	14 (9.9)	65 (9.9)	1.0	796
Insomnia	12 (8.5)	55 (8.7)	1.0	772
Residual malaise	24 (17.0)	90 (14.2)	0.43	775
Laboratory tests at discharge				
BNP, pg/mL	188 (107–557)	179 (77–339)	0.04	511
Hb, g/L	114±20	108±19	<0.001	815
Anaemia	107 (74.8)	546 (81.3)	0.08	815
Alb, g/dL	3.40±0.44	3.28±0.49	0.01	737
Alb <3.0 g/dL	21 (16.8)	140 (22.9)	0.15	737
Na, mEq/L	139.1±3.3	138.8±3.9	0.36	814
Na <135 mEq/L	10 (7.0)	86 (12.8)	0.06	814
K, mEq/L	4.2±0.6	4.3±0.5	0.33	820
K ≥5 mEq/L	10 (7.0)	64 (9.4)	0.42	820
eGFR, mL/min/1.73 m^2^	45.5 (32.1–59.3)	40.5 (27.3–57.5)	0.59	822
eGFR <30 mL/min/1.73 m^2^	32 (22.4)	191 (28.1)	0.18	822
Echocardiography				
LVEF, %	63.6±7.9	62.0±8.0	0.03	829
LVEF >65%	63 (43.8)	224 (32.7)	0.01	829
LVEDD, mm	47.5±6.9	46.0±6.5	0.01	829
LVESD, mm	30.9±6.2	30.1±5.9	0.14	819
LAD, mm	45.1±8.2	41.7±7.0	<0.001	810
LAD >40 mm	103 (74.1)	391 (58.3)	<0.001	810
TRPG, mm Hg	35.9±14.5	32.5±12.5	0.01	656
TR moderate or severe	44 (30.6)	118 (17.4)	<0.001	824
MR moderate or severe	53 (36.8)	156 (23.0)	0.001	821
AS moderate or severe	4 (2.8)	78 (11.5)	<0.001	822
E/A	44.5 (2.6–121)	0.85 (0.67–1.15)	–	830
Medication at discharge				
Loop diuretics	113 (78.5)	518 (75.5)	0.52	830
MRA	61 (42.4)	245 (35.7)	0.15	830
ACEI/ARB	76 (52.8)	396 (57.7)	0.31	830
CCB	47 (32.6)	348 (50.7)	<0.001	830
β-blockers	80 (55.6)	351 (51.2)	0.36	830
Aspirin	48 (33.3)	260 (37.9)	0.34	830
Anticoagulants	61 (42.4)	193 (28.1)	0.001	830
Warfarin	33 (22.9)	96 (14.0)	0.01	830
DOACs	28 (19.4)	97 (14.1)	0.12	830
NSAIDs	2 (1.4)	21 (3.1)	0.40	830
Discharge location				
Home	19 (13.3)	137 (20.0)	0.08	828

Values were expressed as mean±SD, median (IQR) or number with percentage.

Anaemia was diagnosed if the value of Hb was <130 g/L for men and <120 g/L for women.

* Risk adjusting variables selected for the multivariable Cox proportional hazard models.

ACEI, ACE inhibitors; AF, atrial fibrillation; AFL, atrial flutter; Alb, albumin; ARB, angiotensin receptor blockers; AS, aortic stenosis; BMI, body mass index; BNP, brain natriuretic peptide; BP, blood pressure; bpm, beats per minute; CABG, coronary artery bypass grafting; CCB, calcium channel blockers; DOACs, direct oral anticoagulants; DOE, dyspnoea on effort; E/A, early to late diastolic transmitral flow velocity; eGFR, estimated glomerular filtration rate; Hb, haemoglobin; HF, heart failure; HFpEF, HF with preserved ejection fraction; K, potassium; LAD, left atrial diameter; LVEDD, left ventricular end-diastolic diameter; LVEF, left ventricular ejection fraction; LVESD, left ventricular end-systolic diameter; MR, mitral regurgitation; MRA, mineralocorticoid receptor antagonists; Na, Sodium; NSAIDs, non-steroidal anti-inflammatory drugs; NYHA, New York Heart Association; PCI, percutaneous coronary intervention; PND, paroxysmal nocturnal dyspnoea; TR, tricuspid regurgitation; TRPG, tricuspid regurgitation peak gradient.

The date of discharge from the index hospitalisation was regarded as time 0 for clinical follow-up. The cumulative incidence was estimated using the Kaplan-Meier method with intergroup differences assessed by the log-rank test. The Gray method was also used for HF hospitalisation to account for the competing risk of all-cause death. Multivariable Cox proportional hazards models were developed to estimate the HRs and their 95% CIs of patients with restrictive filling pattern relative to those without restrictive filling pattern for the primary and secondary outcome measures. The Fine-Gray subdistribution hazard model was also used for HF hospitalisation to account for the competing risk of all-cause death. To adjust for the confounders, we incorporated the 22 clinically relevant risk-adjusting variables listed in table 1 in accordance with the previous reports.^19 20^ In addition, we performed subgroup analyses stratified by the following parameters to estimate the interactions between subgroup factors and the effect of restrictive filling pattern on clinical outcomes: age ≥80 years, sex, LAD >40 mm, brain natriuretic peptide (BNP) at discharge >181.7 pg/mL (median value), LVEF >65%, prior AF or AFL, valvular heart disease, moderate or severe TR, moderate or severe TR. P values were two-tailed, and statistical significance was set at p<0.05. All data were analysed using EZR software (Saitama Medical Center, Jichi Medical University, Saitama, Japan) or JMP V.18.0.2 software (SAS Institute). Patients and/or the public were not involved in the design, conduct, reporting or dissemination plans of this research.

## Results

### Patient characteristics

**Figure 1:**
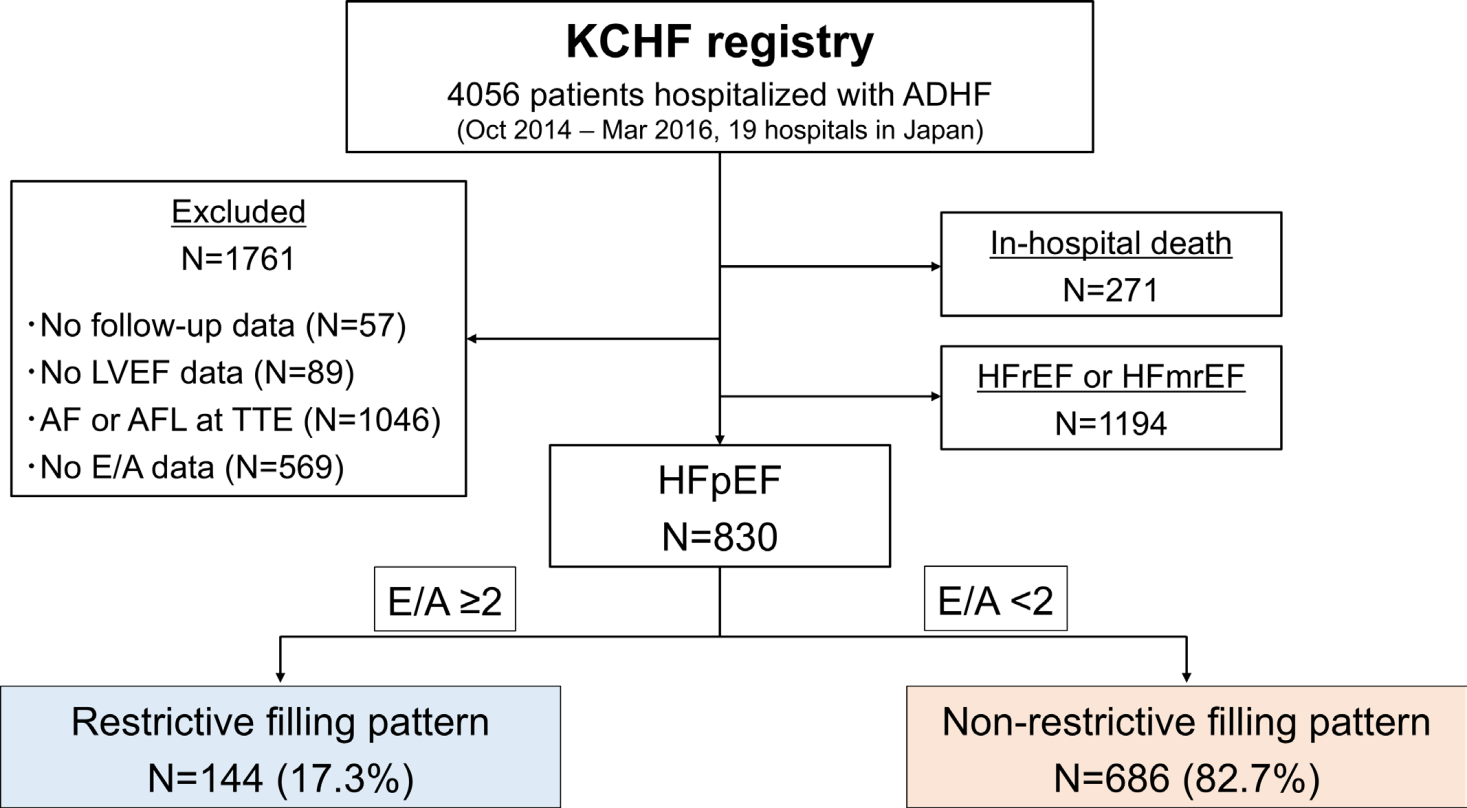
Study patients flow. The study patients were classified according to the presence or absence of restrictive filling pattern with a cut-off value of E/A=2. ADHF, acute decompensated heart failure; AF, atrial fibrillation; AFL, atrial flutter; E/A, early to late diastolic transmitral flow velocity; HFmrEF, heart failure with mildly reduced ejection fraction; HFpEF, heart failure with preserved ejection fraction; HFrEF, heart failure with reduced ejection fraction; KCHF, Kyoto Congestive Heart Failure; LVEF, left ventricular ejection fraction; TTE, transthoracic echocardiography.

Among 830 patients in the current study, there were 144 (17.3%) and 686 (82.7%) patients with E/A≥2 (restrictive filling pattern) and E/A<2, respectively (figure 1). The median time from admission to TTE was 2 (IQR: 0–7) days. The distribution of E/A ratio is shown in online supplemental figure S1. Regarding the patient characteristics, history of prior HF hospitalisation, prior AF or AFL and prescription of anticoagulants were more frequent, while malignancy and prescription of calcium-channel blockers were less frequent in patients with restrictive filling pattern than in those without (table 1). Patients with a restrictive filling pattern had a lower prevalence of sinus rhythm, anaemia and hypoalbuminaemia at admission and at discharge than those without. In addition, patients with a restrictive filling pattern had more residual jugular oedema and higher BNP levels at discharge than those without. As for echocardiographic parameters, the LVEF value and the proportion of supranormal LVEF (>65%) tended to be higher in patients with restrictive filling pattern than in those without. The patient characteristics with and without supranormal LVEF were shown in online supplemental table S1. Patients with restrictive filling pattern were more likely to have larger dimensions of diastolic left ventricle and left atrium, and more often had moderate or severe TR or MR with higher TRPG than those without.

### Clinical outcomes

**Figure 2:**
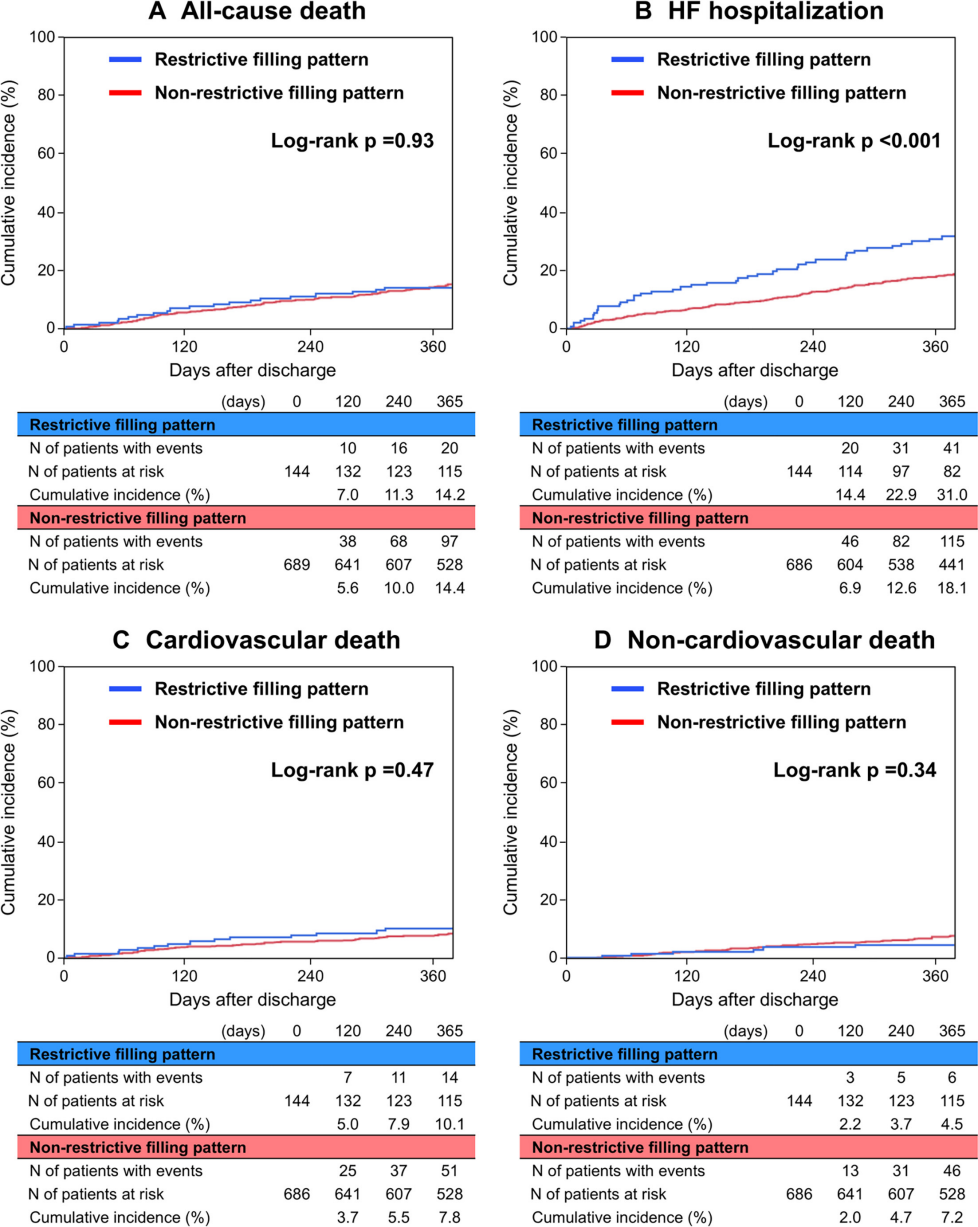
Kaplan-Meier curves according to the presence or absence of restrictive filling pattern for (**A**) all-cause death, (**B**) HF hospitalisation, (**C**) cardiovascular death and (**D**) non-cardiovascular death. HF, heart failure.

The median follow-up duration was 494 (IQR: 373–677) days, with a 92.3% follow-up rate at 1 year. The cumulative 1-year incidence of all-cause death was not different between those patients with and without restrictive filling pattern (14.2% vs 14.4%, p=0.93) (figure 2A). The cumulative 1-year incidence of HF hospitalisation was significantly higher in patients with restrictive filling pattern than in those without (31.0% vs 18.1%, p<0.001) (figure 2B). The cumulative 1-year incidences of cardiovascular death and non-cardiovascular death were not significantly different between the 2 groups of patients with and without restrictive filling pattern (10.1% vs 7.8%, p=0.47; 4.5% vs 7.2%, p=0.34, respectively) (figure 2C, D). After adjusting for confounders, the risk of patients with restrictive filling pattern relative to those without restrictive filling pattern remained not significant for all-cause death (adjusted HR=0.92, 95% CI 0.60 to 1.42, p=0.70). The excess risk of those without restrictive filling pattern remained significant for HF hospitalisation (adjusted HR=1.58, 95% CI 1.10 to 2.27, p=0.01) (table 2). Taking into account the competing risk of all-cause death, restrictive filling pattern was associated with a higher risk for HF hospitalisation (Unadjusted SHR=1.77, 95%CI 1.27 to 2.46, p<0.001; adjusted SHR=1.53, 95%CI 1.06 to 2.19, p=0.02) (online supplemental figure S2 and table S2). In the subgroup analyses, any significant interactions were not found between the clinically relevant subgroup factors and the risk of restrictive filling pattern relative to non-restrictive pattern for all-cause death or HF hospitalisation (online supplemental table S3).

**Table 2:** Clinical outcomes at 1 year

	Restrictive filling pattern		Non-restrictive filling pattern					
	N of patients with events/N of patients at risk	Cumulative incidence	N of patients with events/N of patients at risk	Cumulative incidence	Unadjusted HR (95% CI)	P value	Adjusted HR (95% CI)	P value
Main outcome measure								
All-cause death	20/144	14.2%	97/686	14.4%	0.98 (0.67 to 1.44)	0.93	0.92 (0.60 to 1.42)	0.70
HF hospitalisation	41/144	31.0%	115/686	18.1%	1.77 (1.28 to 2.46)	<0.001	1.58 (1.10 to 2.27)	0.01
Other secondary outcome measures								
Cardiovascular death	14/144	10.1%	51/686	7.8%	1.20 (0.74 to 1.93)	0.47	1.15 (0.66 to 2.00)	0.62
Non-cardiovascular death	6/144	4.5%	46/686	7.2%	0.74 (0.39 to 1.39)	0.34	0.65 (0.32 to 1.32)	0.23

HF, heart failure.

## Discussion

The main findings of the current study were as follows: (1) Restrictive filling pattern was found in 17.3% of patients with HFpEF; (2) Restrictive filling pattern was associated with a significant excess adjusted risk for HF hospitalisation, but not for all-cause death and (3) any significant interactions were not found between the clinically relevant subgroup factors including HF with supranormal ejection fraction (HFsnEF) and the risk of restrictive filling pattern.

HFpEF is not one disease, but a clinical syndrome with highly heterogeneous characteristics. The diagnosis of HFpEF is not always straightforward and sometimes challenging. So far, H2FPEF score and HFA-PEFF diagnostic algorithm, which combined clinical findings and echocardiographic data, have been proposed and validated for the precise diagnosis of HFpEF.^21 22^ HFpEF is characterised by diastolic dysfunction in TTE, and guidelines recommend checking first whether there are reduced e’ velocity (septal ≤6 or lateral ≤7 or average ≤6.5 cm/s), increased E/e’ (septal ≥15 or lateral ≥13 or average ≥14) and increased TR velocity (≥2.8 m/s) or pulmonary artery systolic pressure (≥35 mm Hg) irrespective of LVEF.^12 13^ When LAP is judged to be increased by meeting all three of them or considering other indicators as well, transmitral inflow E/A ratio <2 and ≥2 are classified as grade 2 (mild to moderate increased LAP) and grade 3 (marked increased LAP) diastolic dysfunction, respectively. Thus, the restrictive filling pattern of transmitral inflow, E/A ratio ≥2, has been used as an adjunctive diagnostic tool for diastolic dysfunction. E/A ratio is a highly reproducible and straightforward indicator, but its clinical significance has not yet been clarified in patients with HFpEF.

In the current study, a restrictive filling pattern was observed in 17.3% of HFpEF patients with sinus rhythm at TTE. In a previous report, the prevalence of the restrictive filling pattern was 9% in patients after myocardial infarction with LVEF >53%.^23^ A relatively higher proportion of patients presented a restrictive filling pattern in this study, because the population of the study was elderly, with an average age of 83 years old, and more than 30% of the patients had a history of HF hospitalisation. In patients with a restrictive filling pattern, AF or AFL in the past and/or at admission was more frequent, which was a natural consequence reflecting impaired left atrial function. There was no significant difference in BNP at admission, while BNP at discharge was higher and residual jugular oedema was more often observed in patients with restrictive filling pattern than in those without. Considering BNP in patients with HFpEF was reported to reflect end-diastolic wall stress,^24^ these findings implied that decongestion during hospitalisation became more difficult by diastolic dysfunction in patients with restrictive filling pattern. Regarding echocardiographic findings, larger dimensions of diastolic left ventricle and left atrium, higher TRPG and higher prevalence of moderate or severe TR or MR in patients with restrictive filling pattern were all considered to reflect diastolic dysfunction. Interestingly, the prevalence of supranormal LVEF (>65%) was higher in patients with restrictive filling pattern. HF with LVEF >65% is sometimes classified as HFsnEF. The clinical prognosis of HFsnEF has been reported to be worse than that of HFpEF,^25 26^ although the detailed reason remains unclear. The results of the subgroup analysis did not show significant interaction between HFsnEF and the effects of restrictive filling pattern for clinical outcomes. However, patients with HFsnEF had significantly more cases with restrictive filling pattern, along with smaller LVEDD and LVESD, and a higher TRPG, implying that diastolic dysfunction may be a characteristic finding of HFsnEF.

Previous studies have shown the impact of restrictive filling pattern on clinical outcomes in HFrEF, while data in HFpEF have been scarce. Elena Biagini *et al* reported that restrictive filling pattern in hypertrophic cardiomyopathy (HCM) patients was associated with increased risk of sudden cardiac events, HCM-related death or heart transplantation.^27^ In patients after myocardial infarction and preserved EF, a restrictive filling pattern was also associated with low survival rate.^23^ Our study might extend these observations by demonstrating that, in HFpEF patients hospitalised for acute decompensated HF, restrictive filling pattern was associated with a significantly increased risk of HF rehospitalisation, even after comprehensive adjustment for confounding variables by reflecting advanced diastolic dysfunction or underlying cardiac abnormality. However, the risk for all-cause death was not different between patients with and without restrictive filling pattern. Nevertheless, the cumulative incidence of cardiovascular death was numerically higher in patients with restrictive filling pattern than in those without, which was balanced by the numerically higher incidence of non-cardiovascular death in the former than in the latter. Less non-cardiovascular death in patients with restrictive filling pattern might be explained by a lower prevalence of malignancy, anaemia and hypoalbuminaemia, although the underlying reasons about the difference in patients’ characteristics were currently unknown. The restrictive filling pattern appears to be a sensitive marker of elevated filling pressures and haemodynamic instability that predispose to recurrent decompensation. These findings emphasise the potential value of transmitral inflow assessment as part of the postdischarge risk stratification in HFpEF.

The current study has several limitations. First, this was an observational study and there might be residual confounding factors and selection bias. In addition, the sample size was much smaller than the entire cohort because only patients with HFpEF were included in this study. But to overcome this problem, we adjusted for 22 clinical conceivable factors in the multivariable logistic regression analysis to minimise the confounders. Second, we only analysed transmitral inflow E/A ratio, because we had no data on other diastolic parameters such as peak E-wave velocity, peak A-wave velocity, deceleration time, or tissue Doppler e’ velocity. We would consider conducting research including these parameters in the future. Third, the E/A ratio was solely based on a single TTE performed during hospitalisation. Therefore, it was not possible to analyse the serial data of E/A ratio. Fourth, blood pressure and heart rate at the time of echocardiography were not recorded. In principle, the E/A ratio should be interpreted taking into account blood pressure and heart rate, but we could not perform the analysis incorporating them. Fifth, patients with AF at the time of TTE were excluded from this study as the E/A ratio could not be calculated. AF constitutes a significant component of the aetiology of HFpEF, and excluding these patients might be a major limitation of this study. However, we tried to include as many patients as possible in the analysis, such as including those who had AF at admission but were in sinus rhythm at the time of TTE.

## Conclusions

Restrictive filling pattern of transmitral inflow was associated with an increased risk for HF hospitalisation, but not for all-cause death, in patients with acute decompensated HFpEF.

## Supplementary material

10.1136/openhrt-2026-004093 online supplemental file 1

## Data Availability

Data are available on reasonable request.
