# Supplementary material for: Restrictive filling pattern of transmitral inflow in acute decompensated heart failure with preserved ejection fraction: insights from the KCHF registry
Source: Open Heart. 2026 Apr 17;13(1):e004093. doi: 10.1136/openhrt-2026-004093 (PMC13110694; doi:10.1136/openhrt-2026-004093)
Supplement: online supplemental file 1 [file openhrt-13-1-s001.pdf]

SUPPLEMENTARY MATERIALS

Supplementary Appendix 1. Participating centers and approval number of each participating site

Kyoto University Hospital Kyoto University Graduate School of Medicine (approval number: E2311), Shiga General Hospital (approval number: 20141120-01), Tenri Hospital (approval number: 640), Kobe City Medical Center General Hospital (approval number: 14094), Hyogo Prefectural Amagasaki General Medical Center (approval number: Rinri 26-32), National Hospital Organization Kyoto Medical Center (approval number: 14-080), Mitsubishi Kyoto Hospital (approved 11/12/2014), Okamoto Memorial Hospital (approval number: 201503), Japanese Red Cross Otsu Hospital (approval number: 318), Hikone Municipal Hospital (approval number: 26-17), Japanese Red Cross Osaka Hospital (approval number: 392), Shimabara Hospital (approval number: E2311), Kishiwada City Hospital (approval number: 12), Kansai Electric Power Hospital (approval number: 26-59), Shizuoka General Hospital (approval number: Rin14-11-47), Kurashiki Central Hospital (approval number: 1719), Kokura Memorial Hospital (approval number: 14111202), Kitano Hospital (approval number: P14-11-012), and Japanese Red Cross Wakayama Medical Center (approval number: 328).

Supplementary Appendix 2. Definitions of baseline patient characteristics

Obesity was defined as Body Mass Index (BMI) ≥27.5 kg/m^2 according to the recommendation from WHO Expert Committee for South Asians and Chinese in 2004. The BMI cut-off value of 22 was also used as an ideal index for Asian population. Atrial fibrillation (AF) included paroxysmal AF, persistent AF, and permanent AF. Hypertension was defined as receiving anti-hypertensive drugs or systolic blood pressure ≥140 mmHg or diastolic blood pressure ≥90 mmHg. Diabetes mellitus was defined as treatment with oral hypoglycemic agents and/or insulin, prior clinical diagnosis of diabetes, glycated hemoglobin level ≥6.5 %, casual blood glucose level ≥200 mg/dl, or fasting blood glucose level ≥126 mg/dl. The presence of chronic lung disease was determined clinically by local investigators, based on history, clinical presentation, previous examinations, and medications, recorded as chronic lung disease in the case report form at enrollment. Underlying heart disease was defined as the most likely cause of structural or functional cardiac disorders, among those only one category was chosen. The underlying heart disease was classified as (i) coronary artery disease, (ii) hypertensive heart disease, (iii) cardiomyopathy, (iv) valvular heart disease, (v) other heart diseases. Coronary artery disease was defined as acute coronary syndrome (ACS), old myocardial infarction, or prior percutaneous coronary intervention /coronary artery bypass grafting (PCI/CABG). ACS was defined as the range of myocardial ischemic states that includes ST-elevated myocardial infarction, non-ST elevated myocardial infarction, or unstable angina. Primary cardiomyopathy was classified as hypertrophic cardiomyopathy, dilated cardiomyopathy, and dilated phase of hypertrophic cardiomyopathy. Valvular heart disease was classified as moderate to severe aortic stenosis, aortic regurgitation, mitral stenosis, mitral regurgitation, tricuspid regurgitation, and prosthetic valve dysfunction. As the valvular heart disease, we chose only one category, which seemed to have most closely related to acute heart failure. Other heart diseases included other cardiomyopathy, arrhythmia (bradycardia or tachycardia), congenital heart disease, and constrictive pericarditis. Other cardiomyopathies included arrhythmogenic right ventricular cardiomyopathy, takotsubo cardiomyopathy, cardiac sarcoidosis, cardiac amyloidosis, left ventricular noncompaction, drug-induced cardiomyopathy, pacemaker induced cardiomyopathy, mitochondrial cardiomyopathy, peripartum cardiomyopathy, alcoholic cardiomyopathy, beriberi heart, and others. Chronic kidney disease was defined as an estimated glomerular filtration rate (eGFR) <60 mL/min per 1.73 m^2 at admission. The eGFR was calculated using the equation for the Japanese population: eGFR = 194 × (serum creatinine–1.094) × (age–0.287) × 0.739 (for women). Poor medical adherence was judged by the doctor. Patient's ADL level was assessed

on four level: (1) ambulatory (patients who can walk by themselves), (2) use wheelchair (outdoor only), (3) use wheelchair (outdoor and indoor), (4) bedridden.

Supplementary Table 1. Patient characteristics with and without supranormal LVEF

HFpEF	Supranormal LVEF (+) (LVEF >65%) (N=287)	Supranormal LVEF (-) (LVEF ≤65%) (N=542)	P Value	N of patients analyzed
**Baseline characteristics**				
Age, years	83 [75-88]	82 [74-87]	0.01	829
Age ≥80 years*	184 (64.1)	316 (58.3)	0.12	829
Women*	184 (64.1)	285 (52.6)	0.002	829
BMI, kg/m^2	22.5 [20.3-25.4]	22.5 [20.0-25.3]	0.73	785
BMI (kg/m^2) <22*	122 (43.9)	225 (44.4)	0.94	785
Obesity (BMI (kg/m^2) ≥27.5)	38 (13.7)	73 (14.4)	0.83	785
**Etiology**				
Coronary artery disease	32 (11.2)	95 (17.5)	0.02	829
Acute coronary syndrome*	4 (1.4)	46 (8.5)	<0.001	829
Cardiomyopathy	13 (4.5)	25 (4.6)	1.0	829
Hypertensive heart disease	110 (38.3)	201 (37.1)	0.76	829
Valvular heart disease	90 (31.4)	118 (21.8)	0.003	829
Surgery during hospitalization	6 (2.1%)	9 (1.7%)	0.79	829
Others	42 (14.6)	103 (19.0)	0.12	829
**History**				
Hypertension*	234 (81.5)	436 (80.4)	0.78	829
Diabetes*	89 (31.0)	200 (36.9)	0.09	829

Dyslipidemia	131 (45.6)	200 (36.9)	0.02	829
Prior HF hospitalization*	100 (34.8)	150 (27.7)	0.04	829
Prior PCI or CABG	46 (16.0)	127 (23.4)	0.02	829
Prior myocardial infarction*	20 (7.0)	89 (16.4)	<0.001	829
Prior stroke*	46 (16.0)	92 (17.0)	0.77	829
Prior AF or AFL*	86 (30.0)	146 (269)	0.37	829
Chronic lung disease*	48 (16.7)	77 (14.2)	0.36	829
Malignancy	43 (15.0)	82 (15.1)	1.0	829
Dementia	51 (17.8)	100 (18.5)	0.85	829
Chronic kidney disease	103 (35.9)	249 (45.9)	0.006	829
**Daily life activities**				
Current working	17 (5.9)	60 (11.1)	0.02	829
Not good adherence	47 (16.4)	75 (13.8)	0.35	829
Current smoker*	20 (7.1)	70 (13.1)	0.01	818
Ambulatory	209 (73.1)	418 (78.0)	0.12	822
Living alone*	58 (20.2)	119 (22.0)	0.59	829
**Medications at admission**				
Loop diuretics	140 (48.8)	218 (40.2)	0.02	829
MRA	37 (12.9)	67 (12.4)	0.83	829
ACEI/ARB	150 (52.3)	261 (48.2)	0.27	829
CCB	140 (48.8)	253 (46.7)	0.61	829
β-blockers	95 (33.1)	186 (34.3)	0.76	829

Aspirin	91 (31.7)	180 (33.2)	0.70	829
Anticoagulants	55 (19.2)	103 (19.0)	1.0	829
Warfarin	35 (12.2)	58 (10.7)	0.56	829
DOACs	20 (7.0)	45 (8.3)	0.59	829
NSAIDs	17 (5.9)	45 (8.3)	0.27	829
**Clinical signs at presentation**				
Systolic BP, mmHg	156±35	159±40	0.20	828
Systolic BP <90 mmHg*	7 (2.5)	10 (1.9)	0.61	828
Heart rate, /min	84±27	90±26	0.001	825
Heart rate <60 bpm*	51 (17.9)	50 (9.3)	<0.001	825
NYHA III/IV	245 (86.0)	469 (86.7)	0.83	826
Sinus rhythm	207 (72.1)	403 (74.4)	0.51	829
AF/AFL	49 (17.1)	102 (18.8)	0.57	829
**Laboratory tests at admission**				
BNP, pg/ml	448 [288-844]	607 [318-1134]	0.002	706
Hb, g/dL	10.9±2.2	10.9±2.2	0.96	828
Anemia*	215 (75.2)	410 (75.7)	0.93	828
Alb, g/dL	3.40±0.53	3.45±0.52	0.19	800
Alb <3.0 g/dL*	53 (19.1)	85 (16.3)	0.33	800
Na, mEq/L	139.6±4.3	139.3±4.1	0.42	828
Na <135 mEq/L*	37 (12.9)	59 (10.9)	0.43	828
K, mEq/L	4.1±0.6	4.3±0.7	0.01	828

K ≥5 mEq/L	25 (8.7)	74 (13.7)	0.04	828
eGFR, mL/min/1.73m^2	44.3 [29.7-59.1]	42.0 [27.3-59.2]	0.45	828
eGFR <30 mL/min/1.73m^2*	72 (25.1)	165 (30.5)	0.11	828
**Clinical signs at discharge**				
Systolic BP, mmHg	119±18	122±19	0.03	819
Systolic BP <114 mmHg	114 (40.3)	194 (36.2)	0.26	819
Heart rate, /min	67.5±12.0	69.7±12.6	0.02	816
Heart rate <70/min	172 (61.0)	287 (53.8)	0.05	816
Sinus rhythm	214 (75.1)	428 (80.6)	0.07	816
AF/AFL	43 (15.1)	65 (12.2)	0.28	816
Residual PND	8 (2.9)	27 (5.1)	0.15	803
Residual orthopnea	8 (2.9)	19 (3.6)	0.68	805
Residual DOE	86 (31.1)	121 (23.1)	0.02	801
Residual wheeze	15 (5.4)	19 (3.6)	0.27	801
Residual edema	40 (14.3)	77 (14.7)	0.92	804
Residual jugular edema	20 (7.2)	32 (6.2)	0.55	798
Loss of appetite	32 (11.6)	47 (9.1)	0.26	795
Insomnia	23 (8.5)	44 (8.8)	1.0	771
Residual malaise	42 (15.4)	72 (14.3)	0.67	774
**Laboratory tests at discharge**				
BNP, pg/ml	144 [74-315]	207 [84-380]	0.64	510
Hb, g/dL	10.9±1.9	10.9±1.9	0.81	814

Anemia	220 (78.6)	432 (80.9)	0.46	814
Alb, g/dL	3.31±0.47	3.29±0.49	0.65	736
Alb <3.0 g/dL	52 (20.3)	109 (22.7)	0.51	736
Na, mEq/L	139.1±3.7	138.7±3.8	0.24	813
Na <135 mEq/L	31 (11.0)	65 (12.2)	0.65	813
K, mEq/L	4.2±0.5	4.3±0.5	0.78	819
K ≥5 mEq/L	25 (8.9)	49 (9.1)	1.0	819
eGFR, mL/min/1.73m^2	43.2 [30.7-57.3]	40.2 [26.8-58.8]	0.34	821
eGFR <30 mL/min/1.73m^2	66 (23.3)	157 (29.2)	0.08	821
**Echocardiography**				
LVEF, %	71.3±4.7	57.6±4.6	<0.001	829
LVEDD, mm	44.5±5.8	47.2±6.8	<0.001	828
LVESD, mm	26.6±4.1	32.1±5.9	<0.001	819
LAD, mm	42.0±6.8	42.5±7.7	0.42	809
LAD >40mm	166 (59.5)	328 (61.9)	0.54	809
TRPG, mmHg	34.5±13.7	32.3±12.4	0.04	656
TR moderate or severe	59 (20.7)	103 (19.1)	0.65	823
MR moderate or severe	72 (25.3)	137 (25.6)	0.93	820
AS moderate or severe	34 (11.9)	48 (9.0)	0.18	821
E/A	0.98 [0.72-1.76]	0.92 [0.70-1.46]	0.34	829
E/A ≥2	63 (22.0)	81 (14.9)	0.01	829
**Medication at discharge**				

Loop diuretics	218 (76.0)	412 (76.0)	1.0	829
MRA	90 (31.4)	216 (39.9)	0.02	829
ACEI/ARB	173 (60.3)	299 (55.2)	0.16	829
CCB	135 (47.0)	260 (48.0)	0.83	829
β-blockers	137 (47.7)	294 (54.2)	0.08	829
Aspirin	94 (32.8)	214 (39.5)	0.06	829
Anticoagulants	93 (32.4)	161 (29.7)	0.43	829
Warfarin	50 (17.4)	79 (14.6)	0.31	829
DOACs	43 (15.0)	82 (15.1)	1.0	829
NSAIDs	8 (2.8)	15 (2.8)	1.0	829
**Discharge location**				
Home	48 (16.7)	108 (20.0)	0.26	827

Values were expressed as mean ± standard deviation, median (interquartile range), or number with percentage.

*Risk adjusting variables selected for the multivariable Cox proportional hazard models.

Anemia was diagnosed if the value of hemoglobin was <13 g/dL for men and <12 g/dL for women.

BMI, body mass index; HF, heart failure; PCI, percutaneous coronary intervention; CABG, coronary artery bypass grafting; AF, atrial fibrillation; AFL, atrial flutter; MRA, mineralocorticoid receptor antagonists; ACEI, angiotensin-converting enzyme inhibitors; ARB, angiotensin receptor blockers; CCB, Calcium channel blockers, DOACs, direct oral anticoagulants; NSAIDs, non-steroidal anti-inflammatory drugs; BP, blood pressure; bpm, beats per minute; NYHA, New York Heart Association; BNP, brain natriuretic peptide; eGFR, estimated glomerular filtration rate; PND, paroxysmal nocturnal dyspnea; DOE, dyspnea on effort; LVEF, left ventricular ejection fraction; LVEDD, left ventricular end-diastolic diameter; LVESD, left ventricular end-systolic diameter; LAD, left atrial diameter; TRPG, tricuspid regurgitation peak gradient; TR, tricuspid regurgitation; MR, mitral regurgitation; AS, aortic stenosis.

Supplementary Table 2. Competing risk analysis for HF hospitalization

	Unadjusted SHR [95% CI]	P value	Adjusted SHR [95% CI]	P value
**Restrictive filling pattern**	1.77 [1.27-2.46]	<0.001	1.53 [1.06-2.19]	0.02

SHR, subhazard ratio; CI, confidence interval; HF, heart failure.

Supplementary Table 3. Subgroup analyses for clinical outcomes at 1 year

	Restrictive filling pattern		Non-restrictive filling pattern						
**All-cause death**	**N of patients with events /N of patients at risk**	**Cumulative incidence**	**N of patients with events /N of patients at risk**	**Cumulative incidence**	**Unadjusted HR [95% CI]**	**P value**	**Adjusted HR [95% CI]**	**P value**	**P interaction**
**LA diameter**									
>40mm	14/103	13.9%	58/391	15.1%	1.08 [0.70-1.68]	0.72	0.94 [0.56-1.59]	0.83	0.16
≤40mm	5/36	14.2%	38/280	13.7%	0.63 [0.25-1.58]	0.33	0.57 [0.22-1.52]	0.27	
**BNP at discharge**									
>181.7 pg/ml	8/40	20.2%	37/215	17.4%	0.96 [0.50-1.82]	0.89	1.01 [0.44-2.31]	0.98	0.80
≤181.7 pg/ml	5/35	14.3%	19/221	8.8%	1.03 [0.43-2.47]	0.94	1.36 [0.44-4.18]	0.59	
**LVEF**									
>65%	10/63	16.3%	31/224	14.1%	1.20 [0.68-2.12]	0.54	1.07 [0.55-2.10]	0.84	0.34
≤65%	10/81	12.5%	66/461	14.5%	0.83 [0.49-1.41]	0.49	0.80 [0.43-1.47]	0.47	

**Prior AF or AFL**									
Yes	12/66	18.4%	29/166	17.7%	1.08 [0.61-1.91]	0.79	1.10 [0.56-2.15]	0.78	0.60
No	8/78	10.5%	68/520	13.3%	0.84 [0.49-1.44]	0.52	0.84 [0.45-1.55]	0.57	
**Valvular heart disease**									
Yes	4/35	11.7%	29/173	17.2%	0.80 [0.37-1.72]	0.57	1.52 [0.56-4.07]	0.41	0.84
No	16/109	14.9%	68/513	13.4%	1.05 [0.68-1.64]	0.82	0.92 [0.55-1.51]	0.73	
**Moderate or Severe TR**									
Yes	6/44	13.8%	19/118	16.4%	0.58 [0.28-1.23]	0.15	0.44 [0.17-1.13]	0.09	0.22
No	14/100	14.3%	78/562	14.1%	1.13 [0.72-1.76]	0.61	1.13 [0.68-1.88]	0.64	
**Moderate or Severe MR**									
Yes	7/53	13.5%	16/156	10.5%	1.09 [0.55-2.17]	0.80	2.03 [0.92-4.48]	0.08	0.48
No	13/91	14.6%	80/521	15.6%	0.96 [0.60-1.55]	0.88	0.77 [0.45-1.31]	0.33	
**Age**									
≥80 years	13/82	16.4%	74/419	17.9%	0.91	0.68	0.71	0.20	0.36

					[0.58-1.43]		[0.41-1.21]		
<80 years	7/62	11.3%	23/267	8.8%	1.34 [0.66-2.73]	0.42	1.86 [0.80-4.33]	0.15	
**Sex**									
Female	8/74	11.1%	55/396	14.1%	0.91 [0.54-1.56]	0.74	0.94 [0.50-1.76]	0.84	0.75
Male	12/70	17.4%	42/290	14.7%	1.08 [0.62-1.87]	0.79	0.84 [0.44-1.58]	0.58	
**MRA at admission**									
Yes	5/24	20.8%	12/80	15.1%	1.18 [0.50-2.80]	0.70	2.85 [0.97-8.35]	0.06	0.78
No	15/120	12.8%	85/606	14.3%	0.92 [0.60-1.42]	0.72	0.81 [0.49-1.33]	0.40	
**ACEI/ARB at admission**									
Yes	12/62	19.8%	38/349	11.1%	1.47 [0.87-2.50]	0.15	1.12 [0.59-2.12]	0.74	0.12
No	8/82	9.9%	59/337	17.8%	0.71 [0.41-1.23]	0.22	0.71 [0.38-1.33]	0.29	
**β-blockers at admission**									
Yes	7/46	15.3%	31/235	13.4%	1.17 [0.62-2.20]	0.63	0.76 [0.35-1.65]	0.49	0.44
No	13/98	13.6%	66/451	14.9%	0.89	0.64	0.97	0.91	

					[0.55-1.44]		[0.55-1.70]		

	Restrictive filling pattern		Non-restrictive filling pattern						
**HF hospitalization**	**N of patients with events /N of patients at risk**	**Cumulative incidence**	**N of patients with events /N of patients at risk**	**Cumulative incidence**	**Unadjusted HR [95% CI]**	**P value**	**Adjusted HR [95% CI]**	**P value**	**P interaction**
**LA diameter**									
>40mm	35/103	36.5%	70/391	19.3%	1.92 [1.32-2.79]	<0.001	1.66 [1.08-2.54]	0.02	0.15
≤40mm	5/36	14.8%	43/280	16.8%	1.10 [0.50-2.42]	0.82	1.26 [0.53-2.97]	0.60	
**BNP at discharge**									
>181.7 pg/ml	10/40	27.5%	35/215	17.7%	1.71 [0.92-3.16]	0.09	1.62 [0.75-3.52]	0.22	0.98
≤181.7 pg/ml	6/35	18.8%	29/221	13.9%	1.58 [0.76-3.30]	0.22	1.16 [0.43-3.13]	0.77	
**LVEF**									

>65%	16/63	28.0%	37/224	17.9%	1.82 [1.20-2.75]	0.005	1.29 [0.71-2.33]	0.40	0.73
≤65%	25/81	32.9%	78/461	18.3%	1.73 [1.01-2.97]	0.045	1.60 [1.00-2.55]	0.05	
**Prior AF or AFL**									
Yes	24/66	39.6%	32/166	20.8%	1.90 [1.17-3.08]	0.009	1.84 [1.06-3.18]	0.03	0.48
No	17/78	23.8%	83/520	17.2%	1.44 [0.90-2.31]	0.13	1.44 [0.86-2.43]	0.17	
**Valvular heart disease**									
Yes	11/35	36.2%	27/173	17.4%	1.93 [0.99-3.76]	0.05	1.86 [0.78-4.43]	0.16	0.59
No	30/109	29.5%	88/513	18.3%	1.73 [1.19-2.52]	0.004	1.51 [0.99-2.30]	0.05	
**Moderate or Severe TR**									
Yes	13/44	31.3%	23/118	21.2%	1.44 [0.76-2.76]	0.27	1.10 [0.47-2.59]	0.82	0.60
No	28/100	30.9%	91/562	17.5%	1.90 [1.29-2.78]	0.001	1.86 [1.21-2.86]	0.005	
**Moderate or Severe MR**									
Yes	12/53	24.9%	29/156	19.6%	1.11 [0.60-2.07]	0.73	1.02 [0.45-2.31]	0.97	0.12

No	29/91	34.7%	84/521	17.6%	2.19 [1.49-3.23]	<0.001	1.90 [1.22-2.96]	0.004	
**Age**									
≥80 years	25/82	35.1%	82/419	21.4%	1.59 [1.05-2.43]	0.03	1.37 [0.86-2.18]	0.19	0.43
<80 years	16/62	26.4%	33/267	13.1%	2.21 [1.30-3.74]	0.003	2.51 [1.34-4.68]	0.004	
**Sex**									
Female	19/74	28.5%	67/396	18.3%	1.51 [0.94-2.41]	0.09	1.34 [0.79-2.27]	0.27	0.34
Male	22/70	33.7%	48/290	17.8%	2.09 [1.32-3.31]	0.002	2.08 [1.23-3.55]	0.007	
**MRA at admission**									
Yes	8/24	37.4%	18/80	23.9%	2.02 [0.95-4.33]	0.07	2.60 [0.99-6.78]	0.05	0.98
No	33/120	29.7%	97/606	17.3%	1.71 [1.18-2.46]	0.004	1.49 [0.99-2.24]	0.05	
**ACEI/ARB at admission**									
Yes	17/62	(30.1%)	63/349	(19.1%)	1.62 [0.98-2.68]	0.06	1.70 [0.98-2.94]	0.06	0.24
No	24/82	(31.2%)	52/337	(17.0%)	1.93 [1.24-3.00]	0.003	1.53 [0.91-2.58]	0.11	

**β-blockers at admission**									
Yes	17/46	(39.0%)	52/235	(23.9%)	1.90 [1.17-3.11]	0.01	2.19 [1.22-3.93]	0.008	0.72
No	24/98	(27.1%)	63/451	(15.0%)	1.74 [1.12-2.69]	0.01	1.56 [0.95-2.59]	0.08	

Cumulative incidences at 1 year were indicated as percentages.

HR, hazard ratio; CI, confidence interval; HF, heart failure; LA, left atrium; BNP, brain natriuretic peptide; LVEF, left ventricular ejection fraction; AF, atrial fibrillation; AFL, atrial flutter; TR, tricuspid regurgitation; MR, mitral regurgitation; MRA, mineralocorticoid receptor antagonists; ACEI, angiotensin-converting enzyme inhibitors; ARB, angiotensin receptor blockers.

Supplementary Figure 1. Distribution of E/A ratio

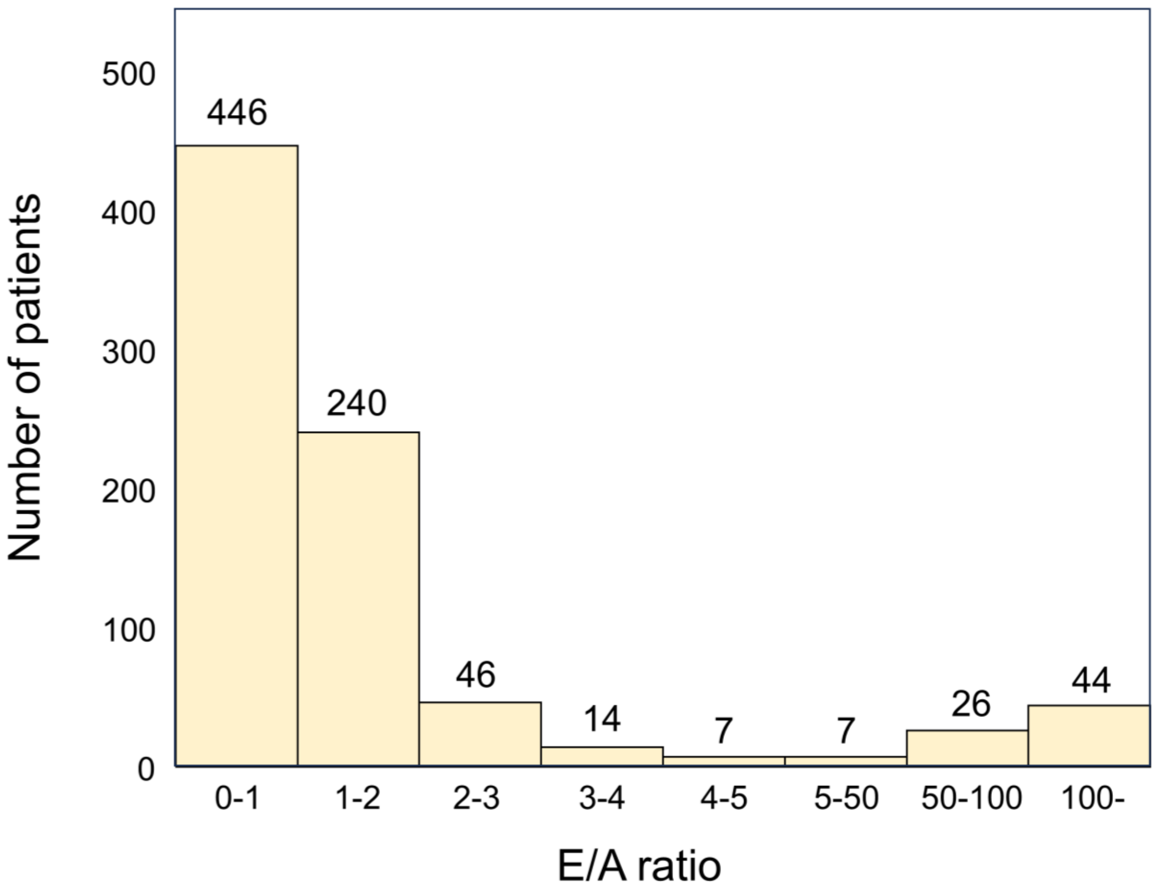

Supplementary Figure 2. Cumulative incidence for HF hospitalization according to the presence or absence of restrictive filling pattern considering competing risk.
HF, heart failure.

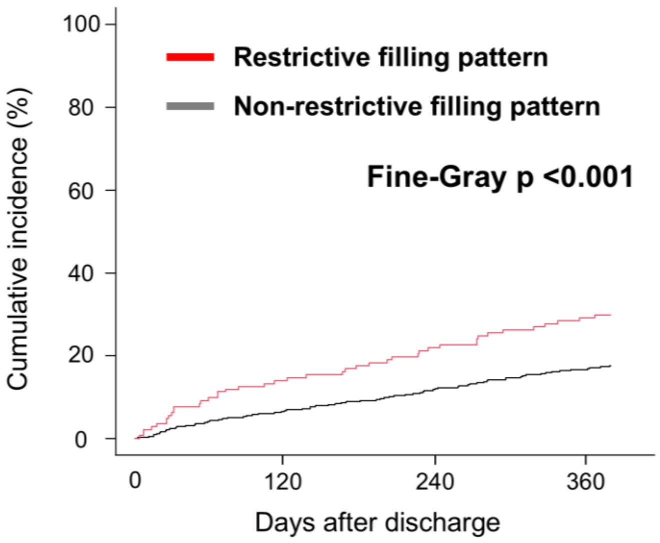

(days)	0	120	240	365
**Restrictive filling pattern**				
N of patients with events		28	42	55
N of patients at risk	144	114	97	82
Cumulative incidence (%)		14.0	21.9	29.2
**Non-restrictive filling pattern**				
N of patients with events		77	138	192
N of patients at risk	686	604	539	435
Cumulative incidence (%)		6.7	12.1	17.0
